# Time of day as a critical variable in biology

**DOI:** 10.1186/s12915-022-01333-z

**Published:** 2022-06-15

**Authors:** Randy J. Nelson, Jacob R. Bumgarner, Jennifer A. Liu, Jharnae A. Love, O. Hecmarie Meléndez-Fernández, Darius D. Becker-Krail, William H. Walker, James C. Walton, A. Courtney DeVries, Brian J. Prendergast

**Affiliations:** 1 Department of Neuroscience, Rockefeller Neuroscience Institute, West Virginia University, Morgantown, WV 26505 USA; 2 Department of Psychology, University of Chicago and Institute for Mind and Biology, IL 60637 Chicago, USA; 3 Department of Medicine, Rockefeller Neuroscience Institute, West Virginia University, Morgantown, WV 26505 USA

**Keywords:** Circadian rhythms, Time of day, Immunology, Neuroscience, Physiology, Pharmacology, Endocrinology and metabolism, Behavioral sciences, Oncology, Cardiac and cardiovascular systems

## Abstract

**Background:**

Circadian rhythms are important for all aspects of biology; virtually every aspect of biological function varies according to time of day. Although this is well known, variation across the day is also often ignored in the design and reporting of research. For this review, we analyzed the top 50 cited papers across 10 major domains of the biological sciences in the calendar year 2015. We repeated this analysis for the year 2019, hypothesizing that the awarding of a Nobel Prize in 2017 for achievements in the field of circadian biology would highlight the importance of circadian rhythms for scientists across many disciplines, and improve time-of-day reporting.

**Results:**

Our analyses of these 1000 empirical papers, however, revealed that most failed to include sufficient temporal details when describing experimental methods and that few systematic differences in time-of-day reporting existed between 2015 and 2019. Overall, only 6.1% of reports included time-of-day information about experimental measures and manipulations sufficient to permit replication.

**Conclusions:**

Circadian rhythms are a defining feature of biological systems, and knowing when in the circadian day these systems are evaluated is fundamentally important information. Failing to account for time of day hampers reproducibility across laboratories, complicates interpretation of results, and reduces the value of data based predominantly on nocturnal animals when extrapolating to diurnal humans.

**Supplementary Information:**

The online version contains supplementary material available at 10.1186/s12915-022-01333-z.

## Background

Platt’s classic distillation of Francis Bacon’s inductive reasoning for the scientific enterprise has served as an essential guideline for generations of scientists [1]. In the model that he termed *strong inference*, Platt outlined four steps to be used for efficient scientific progress in any field: (1) formulate alternative hypotheses, (2) develop a decisive experiment (or a series of experiments) to rule out as many alternative hypotheses as possible, (3) conduct the experiment rigorously to obtain unambiguous results, and (4) recycle the process to test and refine remaining hypotheses [1]. As Platt noted, experiments must be conducted in a rigorous manner.

Experimental studies in biology require rigorous experimental design coupled with sufficiently detailed reporting of methods to allow other scientists to replicate and extend the results. Rigor and reproducibility have become a key initiative at the US National Institutes of Health (NIH) to improve the biomedical scientific enterprise (e.g., NIH guide notice NOT-OD-16–11) [2, 3]. Training in rigor and transparency to increase reproducibility is now mandated for NIH-funded graduate and postdoctoral trainees [4]. Similarly, several scientific societies have revised their publishing guidelines to enhance rigor and reproducibility (e.g., [5–7]). Many of these have focused on providing details of statistical analyses and reagent identification and validation in response to new NIH guidelines. These guidelines require consideration of relevant biological variables, including sex, age, body mass, and underlying health conditions when seeking research funding through the NIH.

Some scientific society guidelines have rightfully called for more comprehensive details of experimental design and analysis in the method sections of published papers to enhance transparency, rigor, and reproducibility [7]. In a recent report, we examined the top 25 most cited papers in several domains of behavioral neuroscience in which previously documented significant time-of-day effects had been reported [8]; remarkably, many of the reviewed studies did not report the time of day when their data were collected (42%), and even when clearly reported, testing was almost as likely to have been performed during the light phase as during the dark phase. The vast majority of animal models used in behavioral neuroscience research are nocturnal rodents; thus, testing during the light phase (i.e., during the animals’ rest period) may confound results and introduce variability across studies [8]. Indeed, it has been reported that dramatic time-of-day effects on neuroprotection in animal models of stroke may contribute to the failures to translate these data to the treatment of stroke and central nervous system diseases in humans [9].

Virtually all physiological and behavioral processes display daily fluctuations driven by endogenous circadian clocks, and the neglect of time-of-day information in methods presents obvious challenges to experimental rigor and reproducibility [10, 11]. In this report, we sought to determine how widespread the failure to report time-of-day information extends beyond behavioral neuroscience. We examined the top 50 cited papers across 10 major domains of the biological sciences in the calendar year 2015. We repeated this analysis for the year 2019, hypothesizing that the awarding of a Nobel Prize in 2017 for achievements in the field of circadian biology would highlight the importance of circadian rhythms for scientists across many disciplines, and improve time-of-day reporting. Time-of-day information was predicted to be more prevalent in the top 50 cited papers in 2019 compared to 2015.

Our analyses, however, revealed that most publications fail to include sufficient temporal details when describing their experimental methods and that there were no differences in time-of-day reporting between 2015 and 2019. Indeed, few studies that we examined included time-of-day factors when interpreting their data. We propose that failing to account for time-of-day as a key biological variable hampers reproducibility across biomedical laboratories, complicates interpretation of the results, and reduces the value of the data when extrapolating laboratory results based on (mainly nocturnal) animal studies to diurnal humans. Below, we review time-of-day reporting data from 10 major discipline areas of the biological sciences: (1) general biology, (2) immunology, (3) neuroscience, (4) physiology, (5) pharmacology and pharmacy, (6) reproductive biology, (7) endocrinology and metabolism, (8) behavioral sciences, (9) oncology, and (10) cardiac and cardiovascular systems. For each area, we (1) briefly highlight the relevance of daily rhythms to core tenets of the discipline and then (2) illustrate, using the 50 most highly cited publications in 2015 and 2019, the patterns of time-of-day reporting in each discipline. Our goal is to raise awareness of the importance of time-of-day as a biological variable that influences reproducibility, reliability, and validity across biological research.

## Results

### Biology

As discussed above, nearly all biological processes display daily fluctuations driven by endogenous circadian clocks. Functional molecular circadian clocks and resultant circadian rhythms in biological processes are fundamental and thus have evolved independently multiple times across all domains of life on planet Earth [12, 13]. Among eukaryotes, the Nobel Prize was awarded for identification of the molecular circadian clock in *Drosophila* [14]. In vertebrates, daily rhythmicity is controlled by a central circadian clock located in the suprachiasmatic nuclei (SCN) in the ventral hypothalamus, which is implicated in virtually all aspects of physiology and behavior (see below). Molecular clocks with similar organizational and functional characteristics, with different molecular mechanisms, have since been identified in all other domains of life. Circadian rhythms in fungi were first described in the genus *Neurospora* in 1959 [15], but the molecular mechanisms of the fungi circadian clock remained unidentified until 1989 [16]. In Neurospora, the molecular circadian clock controls physiology by directing nighttime growth and daytime catabolism [17], and disruption of the molecular clock can directly affect conidiation (reproduction) [18]. Among prokaryotes, circadian rhythms in cyanobacteria were first identified in 1986 [19], but the molecular mechanisms of these clocks remained unidentified for several more years [20, 21]. In these cyanobacteria (*Synechococcus elongatus*) a functioning circadian clock is necessary for natural competence and allows for photoperiodic adaptation [22].

**Fig. 1:**
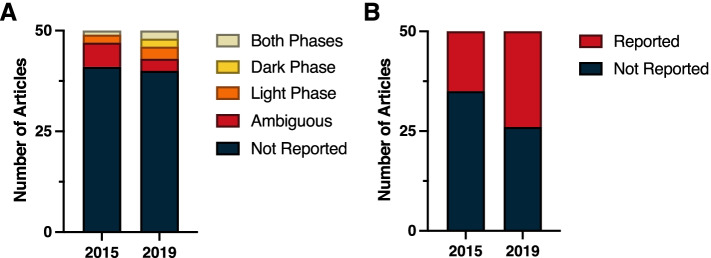
Stack plot indicating the incidence of reporting of (**A**) the time-of-day at which key experimental procedures were performed, and (**B**) experimental lighting cycles among the 50 highest-cited biology reports from 2015 and from 2019. See Methods (Measures and criteria) for time-of-day reporting criteria

Despite the ubiquity of molecular clocks and circadian rhythms in most all living organisms, a review of the top 50 cited papers from general Biology journals in 2015 and 2019 revealed a consistent lack of time-of-day information provided in the methods. In 2015, time-of-day for dependent variables was not reported in 41 of the 50 top cited publications; similarly, 80% (40 of 50) of the top cited publications in 2019 failed to report time-of-day (Fig. 1A) (*χ*^2^ = 1.82; *p* > 0.05) (Additional File 1: Table 1.2). Among papers that did address time-of-day in their methods, this information was ambiguous in six papers in 2015 and in three papers during 2019. Some examples of ambiguity include reporting clock time without reporting time of lights on:off or environmental light schedules, as well as field studies not reporting dates or site location. Only three papers in 2015 and seven papers in 2019 unambiguously identified the time of day for the dependent variables. Likewise, environmental light-dark (LD) cycles were not reported in 35 of 50 of the top cited publications in 2015, nor were they reported in 26 of 50 top publications in 2019 (Fig. 1B) (*χ*^2^ = 3.40; *p* > 0.05) (Additional File 1: Table 1.2). However, some types of general biology studies reviewed here, such as papers describing protein structure or in vitro studies in cells or tissues with no circadian reporters, did not lend themselves for describing, or assuming, time-of-day for their dependent variables. Regardless, time-of-day and environmental photoperiod remain largely ignored as a biological variable, even in these publications that encompass studies across all kingdoms of life.

### Immunology

Circadian rhythms in immune function are ubiquitous and arise from complex interactions among rhythmic features of the host and rhythmic processes intrinsic to immune cells themselves. Daily fluctuations in the numbers of circulating immune cells (e.g., circulating leukocytes or lymphocyte subpopulations) and in their trafficking into and out of the circulation are well-documented in laboratory animals [23–26] and in humans [27, 28]. Innate immune responses to pathogens or pathogen-associated molecules likewise vary over the circadian cycle; in mice and other animal models, the severity of infection symptoms exhibits diurnal patterns [29–32], with more severe responses in animals challenged during the rest phase. Adaptive immune responses, [33] and T cell and B cell functions [34] change over the day, in a species- and trait-specific manner. Circadian clocks within cells of the immune system and circadian clocks distributed more broadly throughout the host interact to drive daily cycles in immune processes [35].

**Fig. 2:**
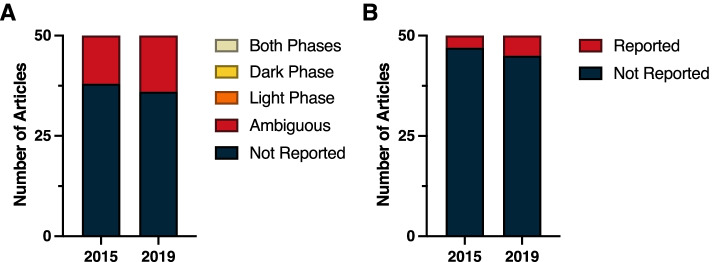
Reporting in immunology. **A** Time-of-day and **B** light cycle reporting

In light of the pervasive effects of circadian time on immune function, it is remarkable that not a single report from either 2015 or 2019 clearly indicated time-of-day information for experimental manipulations and measures (Fig. 2A). In 2015, 12 papers were classified as ambiguous and 38 failed altogether to report time-of-day information; a similar breakdown was evident in 2019 (14 ambiguous, 36 not reported). Reports in the Immunology subdiscipline frequently contained manipulations that were performed in vitro, either in primary culture or on established, immortalized cell lines. Commonly occurring in vitro manipulations, such as temperature pulses and serum delivery, have the potential to induce transcription of circadian clock genes [36–39]. Analogous to information about light–dark cycles, it would be useful to indicate the time at which key experimental manipulations were performed relative to such events.

Finally, vivarium LD cycle information was seldom reported in the Immunology subdiscipline; this information was present in three reports from 2015 and in five reports from 2019 (Fig. 2B) (*χ*^2^ = 0.54; *p* > 0.05) (Additional File 1: Table 1.3).

### Neuroscience

The brain and virtually all aspects of neuroscience exhibit substantial fluctuations across the day. Circadian rhythms and time-of-day variations in physiological function have been well documented within all aspects of brain organization, which is important for synchronizing and adapting behavior to environmental conditions. For example, aspects of cellular and molecular neuroscience, including diurnal synaptic strength and protein markers [40, 41], cortical and motor evoked response, and aspects of behavior including cognition [42], learning and memory [43], and sleep [44] are under clock control and display circadian rhythms. Furthermore, there have also been notable associations between disruptions to circadian rhythms and neurodegenerative diseases, including Alzheimer’s and other related dementias [45], mental health [46], and other neurological disorders [47], suggesting the relevance of time-of-day within translational and clinical neuroscience as well. The subfield of behavioral neuroscience is especially a critical area where the biological variable of time-of-day should be considered [48]. A lack of consistency is prevalent for reporting the timing of behavioral tests; publications fail to consider testing during the animal’s active period, suggesting that not accounting for this variable could affect behavioral outcomes and phenomena [8].

**Fig. 3:**
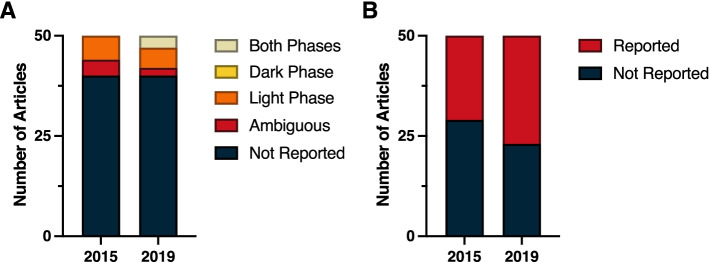
Reporting in neuroscience. **A** Time-of-day and **B** light cycle reporting

Even within this broad multidisciplinary field of Neuroscience, time-of-day as a crucial biological variable often remains unreported. A review of the top 50 cited papers in 2015 and 2019 revealed a substantial lack of time-of-day reporting in the methods. Within both years, time-of-day was not reported in 40 out of the 50 top publications (Fig. 3A) (*χ*^2^ = 0.33; *p* > 0.05) (Additional File 1: Table 1.2). Among papers that listed time of experiments, four papers in 2015 and two papers in 2019 were judged to be ambiguous because they failed to report time of lights on/off for the standard lighting conditions, and also did not report sufficient details to conclude what phase and time the experiments were conducted in relation to circadian time. Circadian time is a standardized 24-h notation of the phase within a circadian rhythm that represents an estimation of individuals’ subjective time. Vivarium light dark cycles were only reported in 29 of the studies in 2015, and 23 of the studies in 2019 (Fig. 3B) (*χ*^2^ = 1.44; *p* > 0.05) (Additional File 1: Table 1.3). However, in vitro studies did not describe or assume time-of-day as a dependent variable. Notably, four studies in 2019 reported circadian timing in the methods section, however, the report of time-of-day as a biological variable often remains neglected in the method sections of neuroscience papers.

### Physiology

The Physiological Society defines physiology as a branch of biology that is distinguished from other physical sciences by an emphasis on the *integration* of molecular, cellular, systems, and whole organism function. Rusak and Zucker [49] published a seminal paper in the *Journal of Physiology* in 1979 that declared rhythms in temporal organization to be comparable to homeostasis in promoting organismal fitness. Michael Rosbash’s 2017 Nobel Lecture [50] emphasized that optimal circadian organization, including temporal coupling or separation of various physiological, metabolic, and behavioral processes, is closely tied to fitness. Given the longstanding emphasis on integration within the field of physiology and acknowledgment that many crucial physiological processes vary across the day, one might reasonably expect publications in the field to emphasize time-of-day information in the methods section.

The integrative approach to physiological research is evident from a perusal of highly cited papers from 2015 and 2019; the vast majority of these papers have both in vivo and in vitro/ex vivo experiments. Among the most common research outcomes of these select papers are inflammation, glucose regulation, blood pressure, generation of reactive oxygen species, and the microbiome. Each of these outcomes has well-characterized circadian rhythms. For example, daily oscillations in gene expression, surface marker expression, cytokine secretion, proliferation, trafficking, effector function, and responsiveness have been described for many immune cells [51, 52]; a proposed adaptive explanation is that circadian regulation of immunity maximizes the benefits of immunity while minimizing the energetic costs and potential secondary damage to tissues (immunopathology) [53]. However, this organization creates a challenge in designing experiments because the peaks and nadirs of various immune cells occur at different points in the circadian cycle. Thus, immunological outcomes may vary significantly based on the time-of-day that the experiment is performed [54]. Similarly, both humans and rodents display daily rhythms in glucose tolerance, with a reduction in tolerance during the rest phase (dark phase in humans and light phase for most rodents) relative to the active phase (light phase in humans and dark phase for most rodents) [55]. Circadian rhythms in glucose availability likely reflect a combination of factors, including diurnal variation in food intake, insulin sensitivity, hepatic glucose production, and pancreatic beta-cell responsivity [55]. Thus, the specific timing of glucose samples, tolerance tests, and assays of processes related to glucose metabolism warrant mention in the “Methods” sections. There are also well-described cardiovascular rhythms in blood pressure and heart rate that tend to follow a 24-h rhythm in which there is a decline during the inactive phase and a rise in anticipation of the start of the active phase (see below and [56]). Not surprisingly, hundreds of genes related to metabolism, signal transduction, and transcription exhibit circadian oscillations in cardiomyocytes [57]. The risk of myocardial infarction follows a similar pattern to blood pressure in humans, with a three-fold increase at the 9:00 h peak compared to the 23:00 h trough [58]. In addition, myocardial infarctions that occur early in the active phase result in larger infarcts [59]. Likewise, in mice, myocardial infarction during the active phase produces larger infarcts and greater deficits in cardiac function than myocardial infarction during the inactive phase due to circadian differences in neutrophil trafficking and resulting inflammation in the myocardium [60]. Lastly, the gut microbiome oscillates in response to several factors including the circadian rhythm of food intake by the host [61], glucocorticoid concentrations, antimicrobial peptide concentrations, and intestinal mucus secretion [62]; there are documented circadian rhythms in microbiome biomass, production of microbially derived products, and gene expression in pathways associated with growth, energy metabolism, motility and detoxification [61, 62]. Together, these studies emphasize the need for precisely timed experimental methods [62].

A common manipulation among the most highly cited physiology papers was exogenous melatonin administration. Melatonin is released from the pineal gland only during the dark phase, is suppressed upon exposure to light, and has potent chronobiotic properties; exogenous administration can phase shift the circadian clock and alter circadian rhythms in endogenous hormones, body temperature, and behavior [63]. Furthermore, time-of-day of administration may influence the physiological response to melatonin [64].

**Fig. 4:**
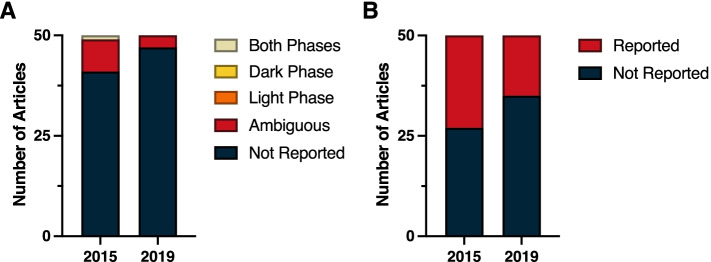
Reporting in physiology. **A** Time-of-day and **B** light cycle reporting

A review of the top 50 cited papers published in the Physiology category in 2015 and 2019 revealed a dearth of time-of-day information provided in the methods (Fig. 4A) (*χ*^2^ = 1.01; *p* > 0.05) (Additional File 1: Table 1.2). The most frequently reported time measure was the number of light versus dark hours in the LD cycle; in 2015, 23 of the 50 papers (46%) reported the LD cycle under which the animals were maintained, whereas in 2019 that number dropped to 15 of the 50 papers (30%; Fig. 4B) (*χ*^2^ = 2.71; *p* > 0.05) (Additional File 1: Table 1.3). Furthermore, 82% of the 2015 papers and 94% of the 2019 papers omitted the time-of-day that specific procedures were performed or samples were collected. Of the nine 2015 papers that provide at least one reference to time of day in the methods, eight were ambiguous (for example, stating that a procedure was performed in the afternoon or providing clock time without sufficient information to convert it to *zeitgbeber* time). Only one of these 2015 papers provided explicit time-of-day information for every procedure and sample collection in the methods section. Among the 2019 papers, three provided ambiguous reporting, with one reporting light phase sleep measurements and another reporting dark phase testing. But, none of these three 2019 papers provided explicit time-of-day information for every procedure and sample collection in the methods section. In sum, only one paper out of the 100 most highly cited papers in 2015 and 2019 within the Physiology category provided sufficient temporal information to allow replication with fidelity to time-of-day.

### Pharmacology and pharmacy

In pharmacology, the efficacy and/or toxicity of a drug is heavily governed by dose, route, and timing of administration. In particular, drug timing has long been known to influence specific drug effects. One of the earliest experiments documenting this phenomenon demonstrated leukemic mice given the same dose of an anti-cancer drug had strikingly different outcomes depending on the time-of-day it was administered; i.e., the same dose yielded few adverse effects when given during the day or inactive phase, but proved lethal when given during the night or active phase [65, 66]. Since then, this circadian variation in drug efficacy or toxicity has been seen in both clinical and preclinical settings, across many classes of compounds and therapies [67]. Accumulating evidence suggests this phenomenon is driven by circadian regulation of the physiology that governs a drug’s pharmacokinetics, now coined chronopharmacokinetics [68].

Chronopharmacokinetics describe a drug’s time-dependent variation across four main processes: absorption, distribution, metabolism, and excretion [69]. Circadian rhythms in gastrointestinal (GI) function directly impact the absorption of a drug, particularly with orally administered compounds (e.g., GI acid secretions, pH, motility, and blood flow all show circadian variation) [70, 71]. Many of the important GI drug influx and efflux transporters show rhythms in expression [72], which may be directly regulated by BMAL1 and the molecular clock [73]. This is also true for many of the hepatic enzymes important for metabolism, including cytochrome P450 (CYP) phase I oxidation metabolism enzymes and multiple phase II conjugation metabolism enzymes [74]. Drug distribution is also heavily dependent on cardiac function, cardiac output, and blood flow, all of which are known to be under circadian regulation and show peaks during the active phase [75–77]. Finally, circadian rhythms in nearly all kidney functions have been documented and contribute to circadian variation in drug excretion [78, 79], a critical last step in limiting a drug’s effects and/or toxicity.

**Fig. 5:**
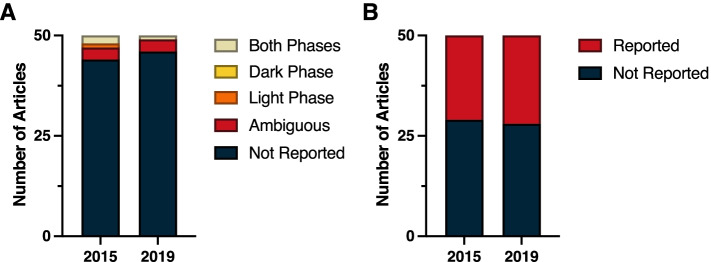
Reporting in pharmacology and pharmacy. **A** Time-of-day and **B** light cycle reporting

Despite extensive literature underscoring the importance of the circadian system in regulating drug effects and/or toxicity, the field of Pharmacology and Pharmacy still largely lacks consistent reporting of time-of-day information. Upon reviewing the top 50 cited papers in 2015 and 2019, the overwhelming majority of papers lack time-of-day reporting. Strikingly, 44 out of 50 in 2015 and 46 out of 50 in 2019 did not report what time-of-day experiments were conducted (Fig. 5A) (*χ*^2^ = 1.04; *p* > 0.05) (Additional File 1: Table 1.2). In both years surveyed, each had three papers that reported time-of-day information, but they were ambiguous or did not report sufficient details to determine the time-of-day experiments were conducted. In 2015, one article reported testing during the light phase and two articles tested across both phases, whereas in 2019 just one article reported testing across both phases. Finally, upon reviewing light cycle reporting, only 21 articles in 2015 and 22 articles in 2019 reported light–dark cycle housing conditions (e.g., 12:12 light–dark cycle); however, none of the articles stated the time of light onset (Fig. 5B) (*χ*^2^ = 0.04; *p* > 0.05) (Additional File 1: Table 1.3).

### Reproductive biology

Reproductive function, from gametogenesis to reproductive behavior, varies across the day [80]. For example, the temporal functioning of ovaries is driven by circadian rhythms arising in the SCN. CLOCK, BMAL1, and CRY1 clock proteins display rhythmic expression in rat ovaries [81]. Clock genes are expressed throughout the ovarian granulosa, theca cells, and luteal cells of rats and other mammals [82]. BMAL1 is significantly elevated ~ 8–10 h after the peri-ovulatory luteinizing hormone (LH) surge at ZT18 on proestrus [81]; female BMAL1 gene knockout mice, however, are infertile likely due to a lack of phasic sensitivity to LH [83].

Importantly, ovulation displays strong time-of-day differences in mammals. For example, rodent ovaries display circadian rhythms of sensitivity to LH; rats injected with equine LH during the dark phase of either diestrus or proestrus ovulated more frequently and produced significantly more oocytes than did females injected during the middle of the light [84]. In contrast, diurnal cattle, gilts, and other female ungulates ovulate during the day [85–87].

Testicular function also displays daily fluctuations [80]. For instance, mice display increased spermatogenesis after the onset of dark compared to mice tested during the early daylight hours [88]. Meiosis of murine spermatids also displays daily rhythms [89]. Daily rhythms of core clock gene and protein expression have been reported in the testes of mice, rats, and hamsters, as well as bulls [90, 91]. There have also been reports of clock gene and protein expression in the epididymis, vas deferens, seminal vesicles, and prostate (reviewed in [90]). In terms of mating behaviors, the vast majority of studies indicate that nocturnal animals mate during the dark (e.g., [92]), whereas diurnal animals such as livestock breed primarily during the day (e.g., [85–87]. Despite these strong circadian rhythms in reproductive biology, the vast majority of highly cited papers ignored time-of-day in their procedural descriptions.

**Fig. 6:**
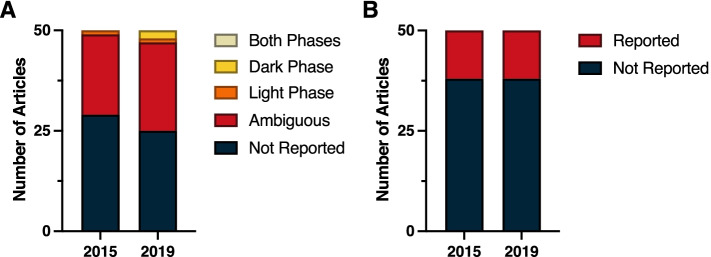
Reporting in reproductive biology. **A** Time-of-day and **B** light cycle reporting

A review of the top 50 cited papers in 2015 and 2019 revealed consistent lack of time-of-day information provided in the methods (Fig. 6A) (*χ*^2^ = 1.04; *p* > 0.05) (Additional File 1: Table 1.2). In many ways, the section on reproductive biology differs from other sections in that diurnal agricultural animals were primarily the topic of the studies, and it was presumed that tissue or other samples were collected during the day. Nonetheless, for many papers the time-of-day information was ambiguous (20 in 2015 and 22 in 2019), but again, inferred to be tested during the day. For example, in many of the highly cited papers in reproductive biology, ovaries or testes were obtained from cattle, swine, sheep, goats, or poultry from local abattoirs presumably during the day shift. In 2015, 29 papers did not report the time-of-day when experimental protocols were conducted, whereas in 2019, 25 papers failed to report time-of-day information (Fig. 6A).

Again, most of the studies in reproductive biology were conducted in livestock, and in 2015 the light cycle information (day length) was not provided in 38 cases. Light cycle information was reported in 12 instances (Fig. 6B). One paper indicated that studies were conducted from February to December 2012 so photoperiod could be determined; two papers indicated that animals were in natural photoperiods, but did not report time of year. In 2019, 38 papers failed to provide light cycle information (Fig. 6B) (*χ*^2^ = 0.0; *p* > 0.05) (Additional File 1: Table 1.3). Twelve of the 50 most cited papers in 2019 reported light cycle information, including one study that collected data in Poland during December so photoperiod could be inferred, but was not explicitly reported.

### Endocrinology and metabolism

Metabolic and endocrine function in animals fluctuates across the day in response to shifts in energy requirements and environmental conditions. As time-of-day variation in food intake occurs, the metabolic systems of animals must adapt in response to varying needs for digestion or mobilization of energy stores [93]. For example, clock genes regulate lipogenesis [94] and glycogenesis [95] across the day in response to temporal variation in food intake. Moreover, the metabolic system also reciprocally interacts with circadian rhythms [96, 97], highlighting the importance of considering time-of-day and circadian rhythms in metabolic research.

Circadian rhythms also regulate hormone production, release, and sensitivity [98]. Numerous hormones display circadian rhythms of secretion, including cortisol, melatonin, growth hormone, ghrelin, and insulin [99]. The rhythms of most hormones differ between nocturnal and diurnal animals, as they help to prepare the body for varying behavioral and physiological needs.

**Fig. 7:**
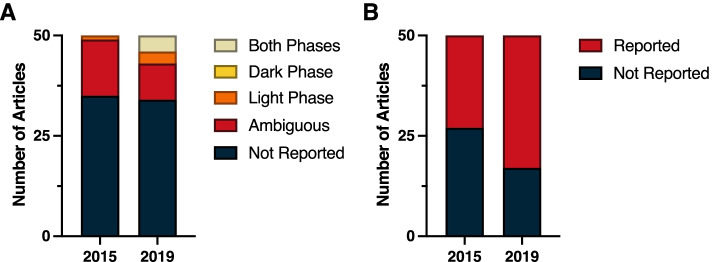
Reporting in endocrinology and metabolism. **A** Time-of-day and **B** light cycle reporting

Over half of the top 50 cited papers from 2015 and 2019 in the field of endocrinology and metabolism did not report the time-of-day at which experiments were conducted; time-of-day reporting improved from 1 out of 50 in 2015 to 7 of 50 in 2019 (Fig. 7A) (*χ*^2^ = 4.89; *p* < 0.05) (Additional File 1: Table 1.2). Fourteen articles from 2015 and 9 articles from 2019 presented ambiguous time-of-day reporting, with a lack of details to sufficiently identify the timing of all experiments conducted. In 2015, only one article reported testing during the light phase, whereas in 2019 three articles reported light-phase testing and four articles conducted experiments across both phases. This improvement was significant based on a chi-squared analysis (*χ*^2^ = 4.89; *p* < 0.05) (Additional File 1: Table 1.2).

In 2015, 27 of the 50 examined articles did not report light–dark cycle housing condition information (Fig. 7B). In 2019, light–dark cycle reporting improved in this field (*χ*^2^ = 4.05; *p* < 0.05) (Additional File 1: Table 1.3), as 33 of the 50 articles provided the hours of vivarium light–dark cycle conditions.

### Behavioral sciences

Daily rhythms in behavior are among the most well-established and well-studied rhythmic phenomena in complex, multicellular animals. Indeed, the discovery of the mammalian circadian pacemaker relied on the high-amplitude circadian rhythm in ingestive behavior of rats—and its elimination via lesions of the so-called master clock, the suprachiasmatic nuclei (SCN) [49, 100]. So robust is the daily cycle of activity and rest that locomotor activity rhythms of mice are leveraged in high-throughput assays for genetic factors that impact the circadian clock [101]. The Behavioral Sciences subdiscipline contained a heterogeneous group of research reports, representing a diversity of species (13 different mammalian species, at least 8 different avian species, 6 fish, 2 lizards, 1 amphibian, 1 insect) and experimental venues (laboratory, field station, research farm, field study). As in many areas of biology, inbred laboratory mice were the modal research model (45 of 100 reports), but rats were commonly featured (24 reports); the linkage in this subdiscipline was an emphasis on behavioral manipulations and measures.

**Fig. 8:**
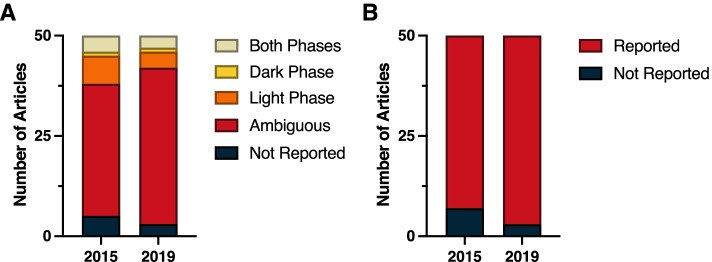
Reporting in behavioral sciences. **A** Time-of-day and **B** light cycle reporting

In contrast to many other subdisciplines surveyed here, LD cycle reporting was common in behavioral sciences (Fig. 8B) (*χ*^2^ = 1.78; *p* > 0.05) (Additional File 1: Table 1.3). In 2015, 43 of 50 (86%) papers clearly reported information on the photocycle exposure, and in 2019, 47 of 50 (94%) reported this information. Collapsing across both survey years, a total of 17 reports exposed animals to natural photoperiods, and 13 of these reports provided information sufficient to infer the photocycle.

In common with other subdisciplines, very few reports indicated the phase of the circadian cycle when experimental manipulations were performed (Fig. 8A) (*χ*^2^ = 1.00; *p* > 0.05) (Additional File 1: Table 1.2). In 2015, time-of-day information was either not reported or ambiguous in over 75% (38 of 50) of the top 50 cited papers, and 2019 yielded a marginally lower ambiguous and non-reporting rate of 84% (42 of 50). A recurring feature across many papers reviewed in this subdiscipline was the use of standard laboratory-based behavioral tests of learning and memory, emotion, attention, and basic sensorimotor function; 55 of the 100 papers surveyed included one or more of these behavioral tests (range: 1–7 tests/report; mode: 3). Only 19 of these 55 (35%) reports indicated time-of-day information for the complete suite of behavioral tests performed: 14 performed testing in the light phase and 5 in the dark phase, although all 19 were conducted using nocturnal rats or mice. Ambiguous or absent reporting for other methodological steps in these 19 papers ultimately required classifying 12 as ambiguous.

Also commonplace among the top cited papers was the collection of blood, brain, feces, or other types of samples for analyses ex vivo. In total, 63 papers reported such collections, but in only 8 instances (13% of reports with collections) was the circadian phase of sample collection made clear. Ambiguous or absent reporting for other methodological steps in these 8 papers ultimately required classifying three as ambiguous.

### Oncology

Of all the described disciplines of animal biology, the field of oncology likely has one of the most integral, but least appreciated, relationship with circadian rhythms. There is a clear bidirectional relationships between core circadian clock genes and cell division [102, 103]. In proliferating mammalian cells, the cell cycle and rhythmic circadian clock are phase-locked [104]. Altered circadian clock gene expression and by consequence altered circadian rhythms are considered a crucial factor for aberrant cell division (i.e., cancer). Indeed, numerous studies have demonstrated distorted clock gene expression in a broad spectrum of cancer types, and foundational sciences studies have demonstrated the functional consequence (i.e., increased cancer growth) of circadian rhythm disruption (reviewed [105]). Given the crucial relationship between circadian rhythms and cancer, it is not unexpected that multiple aspects of cancer biology display time-of-day effects. Indeed, recent studies have demonstrated time-of-day effects in circulating tumor cells [106]. Specifically, circulating tumor cells exhibited stochastic bursts throughout cancer progression with peaks at the onset of the active phase. Additionally, most pertinent to the current review, there is a time-of-day effect in tumor-take frequency following inoculation with cancer cells [107]. Subcutaneous injections of 2000–50,000 fibrosarcoma cells demonstrated a significantly reduced incidence of tumor-take at the sleep/wake transition relative to other times of day. Furthermore, iv injections of the B16 melanoma metastatic cell line exhibited similar time-of-day effects [107]. Time-of-day can also have indirect effects on cancer growth by affecting other systems in the body, for example, the immune system. The immune system displays clear time of day effects (see above), that can have dramatic consequences on cancer development and metastatic spread [108].

**Fig. 9:**
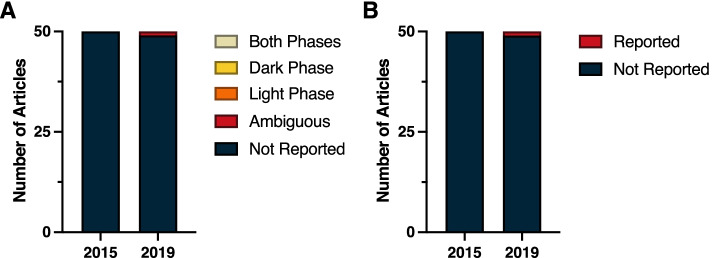
Reporting in oncology. **A** Time-of-day and **B** light cycle reporting

Despite the demonstrated relationship between oncology and circadian rhythms/time-of-day, none of the examined studies provided adequate details in describing the light cycle or timing of experimental manipulations. Of the 100 studies (50 from 2015 and 50 from 2019) examined in the field of oncology, only one study explicitly stated the light dark cycle (Fig. 9B) (*χ*^2^ = 1.00; *p* > 0.05) (Additional File 1: Table 1.3). This was not a consequence of a lack of detailed reporting as some studies mentioned the type of housing, temperature, and humidity, but failed to report the light dark cycle. Furthermore, no study in 2015 described the time-of-day of experimental manipulations, and only one study from 2019 provided an ambiguous description of the time of day of experimental manipulations (i.e., CHK1 inhibitor was injected subcutaneously at the nape of the neck every 12 h; Fig. 9A). The vast majority of studies examined used xenograft or syngeneic tumor models that were then treated. One can assume that virtually all injections, implantations, and treatments likely occurred during the day. However, this is too ambiguous to allow meaningful replication and reproducibility.

### Cardiac and cardiovascular systems

Cardiovascular function also displays daily variations [109, 110] that are regulated by circadian rhythms; cardiovascular function is often linked with sleep–wake patterns [111, 112] and the accompanying shift between sympathetic and parasympathetic innervation (reviewed in [113]). Several core aspects of cardiovascular physiology, such as heart rate and blood pressure variability are key indicators of proper vascular function and reflect predictable day-night fluctuations. Blunting of these circadian fluctuations often coincides with pathological cardiovascular events, such as myocardial infarction, ventricular tachycardia, and sudden cardiac death, peaking in the early morning [114–117]. Further, reduced daily variability of these parameters along with mistimed release of interdependent physiological factors such as endothelial [118, 119], prothrombotic [120], clotting [121], and other core physiological biomarkers, in conjunction with altered immune vulnerability [122], interact to evoke a pathological response [56]. These data suggest circadian regulation over the cardiovascular system. Indeed, peripheral clocks and clock gene expression have been identified in these tissues [123, 124], and differential responses to vasoactive drugs are well established [125, 126]. Thus, time-of-day and circadian phase are important biological variables that need to be considered when choosing animal models to test drugs or other interventions, analyzing and interpreting data, and importantly, making conclusions for potential translatability.

**Fig. 10:**
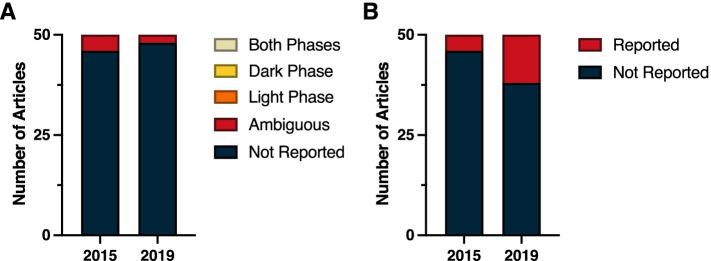
Reporting in cardiac and cardiovascular systems. **A** Time-of-day and **B** light cycle reporting

Despite these well-characterized rhythmic fluctuations in cardiovascular function and physiology, few studies reported time-of-day, environmental light exposure, or circadian rhythm parameters in their research publications. Remarkably, in 2015, only 4 articles reported the animal housing light cycle (12:12) (Fig. 10B) ( *χ*^2^ = 2.99; *p* > 0.05)(Additional File 1: Table 1.2), albeit, without a reference to the time of light onset (zeitgeber time, ZT), compared to 10 articles in 2019, with only one reporting ZT. ZT is a unit of time based on the period of a zeitgeber, such as a light–dark cycle of 12:12 h. In free-running animals housed in constant conditions, the onset of activity of diurnal animals is denoted as circadian time zero (CT0), whereas the onset of activity of nocturnal individuals is CT12. Moreover, none of the 100 articles analyzed for these two years sufficiently detailed the time-of-day (phase) during which experiments were conducted, although it is presumed that these occur during the daytime. Four articles in both 2015 and 2019 had ambiguous reporting, where only partial amounts of reporting were provided or hours of testing were reported without light cycle information.

## Discussion

In this paper, we propose that time-of-day is a crucial biological variable across biological disciplines that should be cited in the methods sections of all published papers. It is well-known, and yet well-ignored, that temporal differences in physiology and behavior exist, and we assert that these time-of-day effects should be considered across all biological studies. For example, an anecdote was described how different results were obtained in the same lab examining a transcription factor. The ‘night owl’ postdoc was able to find this protein (albumin site D-binding protein (DBP)) abundant in hepatocytes, whereas a “morning lark” incoming graduate student could not detect DBP [127]. Similarly, in our lab, we initially reported that balance and motor coordination was unaffected in neuronal nitric oxide synthase (nNOS) knockout mice [128], which seemed inconsistent with reports that the cerebellum possesses the highest numbers of nNOS neurons in the brain. Our initial behavioral phenotyping study was conducted during the day (between 1400 and 1600; lights on at 0700). However, when locomotor behavior was examined during the dark phase, we observed striking differences in balance and motor coordination among the nNOS mice [129].

**Fig. 11:**
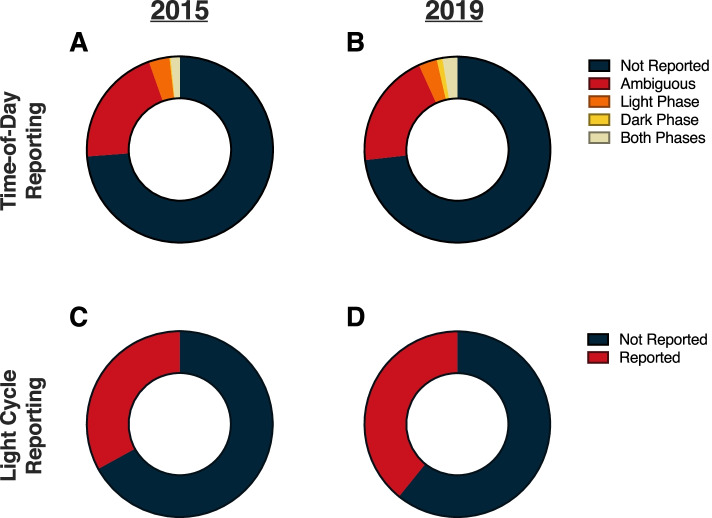
Time-of-day and light cycle reporting across 10 domains of biological sciences. **A, B** Time-of-day and **C, D** light cycle reporting in the top 50 papers across all 10 domains in 2015 and 2019. Each donut chart represents a total of 500 articles

Our analyses in the present paper reveal that information regarding the time-of-day when studies are conducted is routinely omitted from the methods sections of research papers (Fig. 11). Our hypothesis that the 2017 Nobel Prize in Medicine and Physiology that highlighted the importance of circadian rhythms would improve appreciation and reporting of temporal variation in biological systems was not supported overall (*χ*^2^ = 0.86; *p* > 0.05)(Additional File 1: Table 1.2). It is possible that 2 years was insufficient for the Nobel prize to have influenced reporting of time-of-day information in high-impact journal articles because such journals typically have longer editorial, review, and revision cycles. One positive note is that within the field of Endocrinology and Metabolism reporting of time-of-day improved from 2015 to 2019 (*χ*^2^ = 4.89; *p* < 0.05) (Additional File 1: Table 1.2). Reporting of lighting conditions (i.e., light–dark cycles) also improved in this field from 2015 to 2019 (*χ*^2^ = 4.05; *p* < 0.05) (Additional File 1: Table 1.3). Importantly, reporting of light–dark cycles also improved across all fields from 2015 to 2019 (*χ*^2^ = 4.16; *p* < 0.05) (Additional File 1: Table 1.3).

We hope that the present analysis motivates improved reporting of time-of-day as an important biological variable in the future. We contend that this critical biological variable is necessary for reproducible, reliable, and transparent reporting of research.

In an effort to improve the reproducibility of biomedical research, journals are increasingly requiring submissions that involve animal research to adhere to minimum standards of methodological reporting. One such set of guidelines, the ARRIVE guidelines, includes information on sample sizes, randomization, blinding, statistical models, and results reporting, along with experimental procedures [130]. In this latter category, ARRIVE guidelines recommend information on what, how, where, why, and when procedures were performed, specifically that “Methods” sections: “Clearly report the frequency and timing of experimental procedures and measurements, including the light and dark cycle, circadian time cues, and experimental time sequence” [130]. It appears that very few journals require reporting of time-of-day information. This appears to be a joint failure of journals and reviewers to check that the ARRIVE guidelines are being enforced. We recommend that the ARRIVE guidelines should be enforced regarding time-of-day for experimental methods going forward to improve reliability and replicability.

Again, we assert that time-of-day is a controllable and critical biological factor that should be considered in the design, implementation, and analyses of experimental data. Importantly, time-of-day of animal testing, sample collection, as well as in vitro tests must be tightly controlled and described in detail. In some cases, it may be necessary to test during the light phase. For example, the use of some automated behavioral testing tools often requires animals to be tracked in the light. Nonetheless, to improve time-of-day reporting, details regarding time-of-day, photoperiod, time of testing (either clock time, circadian time, or zeitgeber time), and whether testing occurred during the dark or light should always be reported in every methods section. If testing occurs during the dark, then methods for protecting circadian rhythms such as using dim red lighting or night vision goggles must be described.

## Conclusion

In sum, consideration of circadian rhythms across biological studies is critical to enhance experimental rigor and reproducibility and crucial for the interpretation of study results. Life on Earth is adapted to the 24-h solar day and adaptations to temporal niches have shaped virtually all aspects of biology during evolution to increase fitness. Ignoring these temporal influences during the conduct of animal studies influences the collected data, and muddles interpretation. Together, evidence-based decision-making in the timing of data collection, protection against exposure to extraneous light during dark phase testing, incorporation of temporal factors in data analysis and interpretation, and meticulous reporting of temporal factors in publications, have the potential to improve experimental rigor and reproducibility across all fields in biology.

## Methods

### Data sources

Using Web of Science, we examined the top cited 50 papers in several domains of biology for 2015 and 2019 to determine what time-of-day experiments were conducted or whether time of day was reported. These domains comprise: (1) general biology, (2) immunology, (3) neurosciences, (4) physiology, (5) pharmacology and pharmacy, (6) reproductive biology, (7) endocrinology and metabolism, (8) behavioral sciences, (9) oncology, and (10) cardiac and cardiovascular systems. Using Web of Science category domain searches with the results sorted by highest citation counts and examined studies that included living non-human animals in any aspect of the experimental design. Article duplications between the fields were also examined, and were found to be minimal (17 duplicated articles/1000 articles)(Additional File 2: Fig. 1A-B). We excluded studies that exclusively examined humans, computational studies without animal data, or review papers from our analyses.

### Measures and criteria

We recorded the species used in each study, the datapoints collected and reported, the experimental manipulations, and whether the study was in vivo, in vitro, or ex vivo. The principal data abstracted from each report were (1) indication of the circadian phase during which experimental manipulations were performed (i.e., light, dark, both, not reported) and (2) information about the vivarium photocycle under which experimental animals were tested.

Experimental manipulations were coded as being performed in the “light phase,” in the “dark phase,” or in “both phases,” if clear and consistent reporting was available for all procedures; we also noted whether circadian (zeitgeber) time was indicated for procedures. We categorized temporal information as “ambiguous” if clock time was stated without providing the light–dark cycle information or if the information could be inferred, but was not explicitly stated. We also categorized reports as “ambiguous” if circadian phase information was provided for some, but not all, key manipulations/procedures. Examples of “key” manipulations/procedures include behavioral tests, blood sampling, and tissue collection. The widespread practice of merely citing published methods in the description of an experimental manipulation was not regarded as adequate for the purposes of indicating vivarium LD cycle information or time-of-day information.

Vivarium photocycle information was coded as “reported” or “not reported” if such information was provided. If animals were exposed to natural photoperiods, then photocycle information was considered to be available if it could be determined from the latitude and time of year at which the experiment was performed.

Prevalent among the reports surveyed were common behavioral assays of learning and memory (Morris water maze, Barnes test, conditioned place preference, fear conditioning, spontaneous alternation, novel object recognition, avoidance tasks) emotion-like behavior (spontaneous locomotion, open field, elevated plus, tail suspension, marble burying, forced swim, tail suspension, sucrose preference, splash test, social motivation), motor function (limb strength, Rotarod) and attention (pre-pulse inhibition), many of which use bright light as a component of the motivational paradigm, but may also be performed under conditions of darkness. Because these tests may be performed in the light or dark phase of the circadian cycle, and consistent with prior analyses [8], we classified these tests as “ambiguous” if they failed to report time of testing, provided time of testing for some, but not all tests, or provided the time of testing without linkage to a specific phase of the LD cycle. In addition, many of the top cited papers performed collection of blood, brain, feces, and other types of samples for analyses ex vivo. Because such collections may be readily performed during the light or dark phase, unless information about the phase of collection was available, these procedures were coded as “not reported.”

Finally, although it may be reasonable to assume that many other manipulations and dependent measures were collected during the typical workday, which commonly overlaps with the light phase of the experimental animals, many of these same procedures can also be performed during the dark phase. Moreover, many laboratories house animals in reversed light–dark cycles. Thus, with few exceptions (discussed in the “Results” above), if time-of-day information was not explicitly available, such measures were coded as “not reported.”

## Supplementary Information

**Additional file 1.**

**Additional file 2.**

## Data Availability

All data generated or analyzed during this study are included in this published article and its supplementary information files.
