# Supplementary material for: Time of day as a critical variable in biology
Source: BMC Biol. 2022 Jun 15;20:142. doi: 10.1186/s12915-022-01333-z (PMC9202143; doi:10.1186/s12915-022-01333-z)
Supplement: Supplementary file 1 — Additional file 1. [file 12915_2022_1333_MOESM1_ESM.pdf]

Summary Table 1.1

		Time of Day Reporting					Light-Dark Cycle Reporting	
**Domain**	**Year**	**Not Reported**	**Ambiguous**	**Light**	**Dark**	**Both**	**Not Reported**	**Reported**
Behavioral Sciences	2015	5	33	7	1	4	7	43
Behavioral Sciences	2019	3	39	4	1	3	3	47
Biology	2015	41	6	2	0	1	35	15
Biology	2019	40	3	3	2	2	26	24
Cardiac and Cardiovascular Systems	2015	46	4	0	0	0	46	4
Cardiac and Cardiovascular Systems	2019	46	4	0	0	0	40	10
Endocrinology and Metabolism	2015	35	14	1	0	0	27	23
Endocrinology and Metabolism	2019	34	9	3	0	4	17	33
Immunology	2015	38	12	0	0	0	47	3
Immunology	2019	36	14	0	0	0	45	5
Neuroscience	2015	40	4	6	0	0	29	21
Neuroscience	2019	40	2	5	0	3	23	27
Oncology	2015	50	0	0	0	0	50	0
Oncology	2019	49	1	0	0	0	49	1
Pharmacology and Pharmacy	2015	44	3	1	0	2	29	21
Pharmacology and Pharmacy	2019	46	3	0	0	1	28	22
Physiology	2015	41	8	0	0	1	27	23
Physiology	2019	47	3	0	0	0	35	15
Reproductive Biology	2015	29	20	1	0	0	38	12
Reproductive Biology	2019	25	22	1	2	0	38	12
	**Total 2015**	369	104	18	1	8	335	165
	**Total 2019**	366	100	16	5	13	304	196
	Total % with sufficient reporting:			**6.1%**			**TOD**	**LD**
					2015 Paper Count:		500	500
					2019 Paper Count:		500	500

Summary Table 1.2

Time of Day Reporting Chi-Squared Analysis Domain-by-Domain		Phase Reporting		Expected Values (Row_sum*Column_Sum) /Table_sum		Chi-Squared Terms (O-E)^2/E				
**Domain**	**Year**	**Sufficient**	**Insufficient**	**Sufficient**	**Insufficient**	**Sufficient**	**Insufficient**	**X^2**	**P**	**Different Reporting?**
**Behavioral Sciences**	2015	12	38	10	40	0.4	0.1	1	0.31731051	No
	2019	8	42	10	40	0.4	0.1			$p > 0.05$
**Biology**	2015	3	47	5	45	0.8	0.08888889	1.77777778	0.18242244	No
	2019	7	43	5	45	0.8	0.08888889			$p > 0.05$
**Cardiac and Cardiovascular Systems**	2015	0	50	Chi-squared not possible, column with zeros						
	2019	0	50							
**Endocrinology and Metabolism**	2015	1	49	4	46	2.25	0.19565217	4.89130435	0.02699229	*** Yes**
	2019	7	43	4	46	2.25	0.19565217			**$p < 0.05$**
**Immunology**	2015	0	50	Chi-squared not possible, column with zeros						
	2019	0	50							
**Neuroscience**	2015	6	44	7	43	0.14285714	0.02325581	0.33222591	0.56435132	No
	2019	8	42	7	43	0.14285714	0.02325581			$p > 0.05$
**Oncology**	2015	0	50	Chi-squared not possible, column with zeros						
	2019	0	50							
**Pharmacology and Pharmacy**	2015	3	47	2	48	0.5	0.02083333	1.04166667	0.30743417	No
	2019	1	49	2	48	0.5	0.02083333			$p > 0.05$
**Physiology**	2015	1	49	0.5	49.5	0.5	0.00505051	1.01010101	0.31487864	No
	2019	0	50	0.5	49.5	0.5	0.00505051			$p > 0.05$
**Reproductive Biology**	2015	1	49	2	48	0.5	0.02083333	1.04166667	0.30743417	No
	2019	3	47	2	48	0.5	0.02083333			$p > 0.05$

Time of Day Reporting Chi-Squared Analysis Domain-by-Domain		Phase Reporting		Expected Values (Row_sum*Column_Sum) /Table_sum		Chi-Squared Terms (O-E)^2/E				
**Domain**	**Year**	**Sufficient**	**Insufficient**	**Sufficient**	**Insufficient**	**Sufficient**	**Insufficient**	**X^2**	**P**	**Different Reporting?**
**Overall**	2015	27	473	30.5	469.5	0.40163934	0.02609159	0.85546186	0.35501178	No
	2019	34	466	30.5	469.5	0.40163934	0.02609159			$p > 0.05$

Summary Table 1.3

Light-Cycle Reporting Chi-Squared Analysis Domain-by-Domain		Phase Reporting		Expected Values (Row_sum*Column_Sum) /Table_sum		Chi-Squared Terms (O-E)^2/E				
**Domain**	**Year**	**Sufficient**	**Insufficient**	**Sufficient**	**Insufficient**	**Sufficient**	**Insufficient**	**X^2**	**P**	**Different Reporting?**
**Behavioral Sciences**	2015	43	7	45	5	0.08888889	0.8	1.77777778	0.18242244	No p > 0.05
	2019	47	3	45	5	0.08888889	0.8			
**Biology**	2015	15	35	19.5	30.5	1.03846154	0.66393443	3.40479193	0.06500731	No p > 0.05
	2019	24	26	19.5	30.5	1.03846154	0.66393443			
**Cardiac and Cardiovascular Systems**	2015	4	46	7	43	1.28571429	0.20930233	2.99003322	0.08377845	No p > 0.05
	2019	10	40	7	43	1.28571429	0.20930233			
**Endocrinology and Metabolism**	2015	23	27	28	22	0.89285714	1.13636364	4.05844156	0.04395104	*** Yes p < 0.05**
	2019	33	17	28	22	0.89285714	1.13636364			
**Immunology**	2015	3	47	4	46	0.25	0.02173913	0.54347826	0.46099479	No p > 0.05
	2019	5	45	4	46	0.25	0.02173913			
**Neuroscience**	2015	21	29	24	26	0.375	0.34615385	1.44230769	0.22976627	No p > 0.05
	2019	27	23	24	26	0.375	0.34615385			
**Oncology**	2015	0	50	0.5	49.5	0.5	0.00505051	1.01010101	0.31487864	No p > 0.05
	2019	1	49	0.5	49.5	0.5	0.00505051			
**Pharmacology**	2015	21	29	21.5	28.5	0.01162791	0.00877193	0.04079967	0.8399251	No p > 0.05
	2019	22	28	21.5	28.5	0.01162791	0.00877193			
**Physiology**	2015	23	27	19	31	0.84210526	0.51612903	2.71646859	0.09931753	No p > 0.05
	2019	15	35	19	31	0.84210526	0.51612903			
**Reproductive Biology**	2015	12	38	12	38	0	0	0	1	No p > 0.05
	2019	12	38	12	38	0	0			

Light-Cycle Reporting Chi-Squared Analysis Domain-by-Domain		Phase Reporting		Expected Values (Row_sum*Column_Sum) /Table_sum		Chi-Squared Terms (O-E)^2/E				
**Domain**	**Year**	**Sufficient**	**Insufficient**	**Sufficient**	**Insufficient**	**Sufficient**	**Insufficient**	**X^2**	**P**	**Different Reporting?**
**Overall**	2015	165	335	180.5	319.5	1.33102493	0.75195618	4.16596222	0.04124398	*** Yes p < 0.05**
	2019	196	304	180.5	319.5	1.33102493	0.75195618			
