# Supplementary figures and images for: Time of day as a critical variable in biology
Source: BMC Biol. 2022 Jun 15;20:142. doi: 10.1186/s12915-022-01333-z (PMC9202143; doi:10.1186/s12915-022-01333-z)

**2015**

**A**

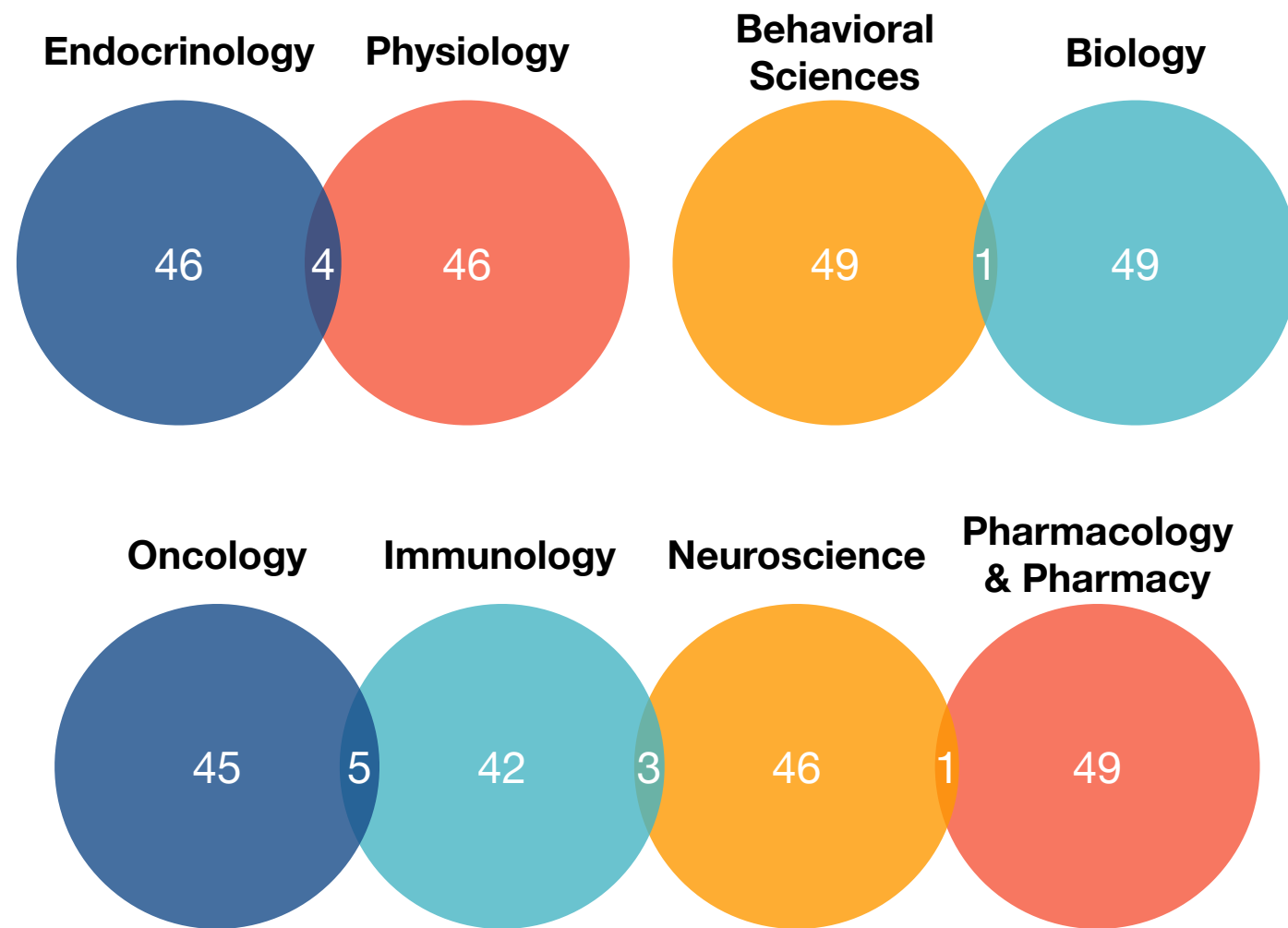

**2019**

**B**

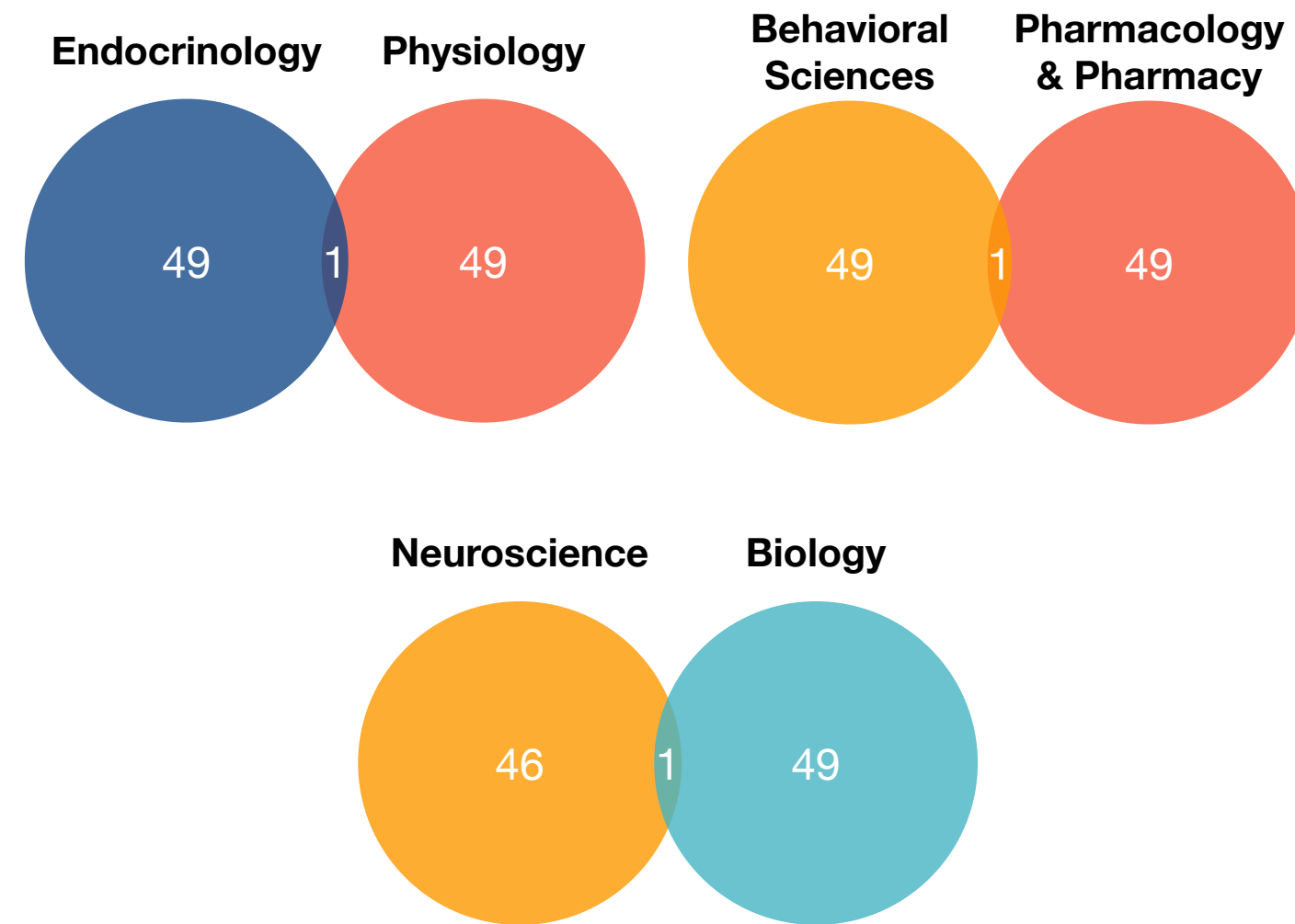

Supplement: Supplementary file 2 — Additional file 2. [file 12915_2022_1333_MOESM2_ESM.pdf]
